# Type 1 Diabetes Mellitus Pathogenesis: Mechanisms, Early Diagnostic Strategies, and Emerging Therapeutic Approaches

**DOI:** 10.3390/pathophysiology33030048

**Published:** 2026-07-09

**Authors:** Nokwanda N. Ngcobo, Ntethelelo H. Sibiya

**Affiliations:** 1 Discipline of Pharmaceutical Sciences, School of Health Sciences, Durban 4001, South Africa; 2 Pharmacology Division, Faculty of Pharmacy, Rhodes University, Makhanda 6139, South Africa; n.sibiya@ru.ac.za

**Keywords:** type 1 diabetes, autoimmunity, triggers, gut dysbiosis, viral infections, obesity, beta cell

## Abstract

Type 1 diabetes mellitus (T1DM) is regarded as an autoimmune disorder characterized by a progressive loss of β-cells, culminating in insulin deficiency and hyperglycemia. Currently, T1DM is managed through exogenous insulin administration. Continued efforts to decipher T1DM pathogenesis have yielded significant progress, which can be harnessed to develop disease-modifying therapeutic modalities. Immunological and genetic studies have demonstrated pathogenetic mechanisms that underpin the sensitivity and risk of developing autoimmunity, ultimately leading to the destruction of β-cells. Genetic and immunological studies, therefore, suggest that certain individuals are at risk of developing T1DM; and that autoantibodies against β-cells develop and circulate years before the onset of symptomatic T1DM. Furthermore, the onset of autoimmunity has been associated with specific triggers in genetically susceptible individuals. Recent developments have revealed how viral infections, gut dysbiosis, dietary factors, and obesity trigger autoimmunity and β-cell damage. In this review, we present the current, consolidated understanding of T1DM pathogenesis, informed by recent research. We further identify opportunities for early-detection strategies and drug development targeting the asymptomatic phase of T1DM to slow disease progression. Currently, immunomodulatory strategies have yielded promising outcomes in clinical trials. These strategies seek to target immune cells and inflammatory mediators implicated in the pathogenesis of T1DM. Other strategies include tolerogenic strategies against autoimmune cells, whilst others employ β-cell protection. The emergence of regenerative therapies also offers promising avenues toward T1DM.

## 1. Introduction

Type 1 diabetes mellitus (T1DM) is an autoimmune disorder characterized by immune-mediated destruction of pancreatic β-cells, resulting in insulin deficiency and chronic hyperglycemia. The disease has been historically viewed predominantly as an insulin-deficient state [1]. Emerging evidence increasingly reframes T1DM as a heterogeneous, multihormonal, and immunometabolic disorder driven by intricate interactions among immune pathways, β-cell intrinsic stress, genetic susceptibility, environmental exposures, and broader endocrine dysfunction [2,3]. Traditionally, its pathophysiology is explained primarily through human leukocyte antigen (HLA) linked genetic susceptibility and autoantibody development. However, recent advances have refined our understanding, positioning T1DM as a heterogeneous syndrome driven by complex genetic, environmental, immunological, and β-cell-intrinsic factors [4,5,6]. Recent syntheses emphasize that classical HLA-linked risk interacts with many non-HLA loci, and that diverse immune effectors (such as autoreactive CD4+ and CD8+ T cells, B cells, and innate immune elements) contribute to β-cellinjury and the variable pace of disease progression [7,8].

Newer high-resolution immunophenotyping and single-cell studies have clarified that islet autoimmunity involves multiple T-cell clones and tissue-resident features of insulitis. This may help explain why some individuals progress rapidly while others exhibit long preclinical phases. These mechanistic insights have catalyzed innovative prevention strategies, shifting T1DM from an inevitably progressive disease toward one in which early detection and intervention may delay or even prevent onset [9,10,11,12].

Understanding of pathogenesis has shifted attention toward β-cell fragility and intrinsic β-cell “stress” as co-drivers (not merely passive victims). Recent studies have highlighted that metabolic challenge, viral exposures, and local inflammation can alter β-cell antigen expression and promote the development of neoantigens, thereby amplifying autoimmune recognition [13,14,15]. This reframes therapeutic goals toward preserving “stressed” β-cells and reducing antigen presentation, strategies that may be as important as broadly suppressing immune cells. This review aims to consolidate developments toward the pathophysiological understanding of T1DM. We envisage that through this exercise, opportunities for strategic pharmacological targets for intervention could be identified, with the goal of preventing or delaying the onset of T1DM.

## 2. Methodology

This narrative review explores recent advances in understanding the pathophysiology of T1DM, with particular emphasis on opportunities for early detection and prevention. Relevant peer-reviewed literature published in English between 2010 and 2026 was identified through a comprehensive literature search of PubMed, ScienceDirect, and Scopus using the following search terms: type 1 diabetes, autoimmunity, environmental triggers, gut dysbiosis, viral infections, and obesity. The reference lists of eligible articles and key review papers were also screened to identify additional relevant studies that may not have been captured in the database search.

Article selection followed a structured, multi-step process. Titles and abstracts were initially screened for relevance to T1DM pathophysiology, early detection, or prevention. Full-text articles were subsequently retrieved and assessed against predefined inclusion and exclusion criteria. Studies were included if they addressed advanced or emerging aspects of T1DM pathophysiology, were published in English, and discussed at least one aspect of early detection or prevention strategies. Articles focused exclusively on T1DM management or treatment, without discussion of pathophysiological mechanisms, detection, or prevention, were excluded. Conference abstracts, non-peer-reviewed publications, and studies lacking sufficient methodological detail were also excluded.

Priority was given to systematic reviews and meta-analyses as the highest level of synthesized evidence, followed by randomized controlled trials and large prospective cohort studies. Landmark studies were included irrespective of publication date when they provided foundational mechanistic insights into T1DM development. More recent mechanistic studies (2018–2026) were prioritized to reflect advances in understanding autoimmune triggers, immune dysregulation, genetic susceptibility, and the gut microbiome’s role. Where clinical trial evidence was limited, well-designed observational studies and expert consensus statements were considered to provide a comprehensive overview of the available evidence.

Conflicting evidence was evaluated and presented transparently throughout the review. Where studies reported divergent findings, particularly regarding environmental triggers, dietary factors, or viral contributions to T1DM onset, differences were interpreted in the context of study design, population characteristics, follow-up duration, diagnostic criteria, and methodological quality. Rather than imposing consensus, areas of uncertainty and ongoing scientific debate were highlighted to provide a balanced assessment of the current evidence base and to identify priorities for future research.

The findings were synthesized using a thematic analysis and organized into key pathophysiological domains: genetic susceptibility, immune mechanisms, environmental and microbial triggers, and metabolic interactions relevant to disease initiation and progression.

## 3. Genetic and Immunological Basis of Pathogenesis

The pathogenesis of T1DM requires the convergence of genetic susceptibility and additional, as yet incompletely defined factors. Genetic susceptibility involves both HLA and non-HLA loci [16]. The HLA genes are particularly crucial because they determine which foreign substances the immune system recognizes as threats. Certain HLA variants significantly increase the risk of T1DM by making the immune system more likely to mistakenly attack the body’s own pancreatic β-cells. However, genetics alone are insufficient to trigger the disease. HLA class II alleles, principally HLA-DR and HLA-DQ, account for approximately 50% of familial clustering and confer the strongest known genetic risk [17,18]. Specific haplotype combinations are associated with a spectrum of risk levels, ranging from high susceptibility to relative protection [19]. Non-HLA loci, including INS, PTPN22, and CTLA4, contribute additional, more modest risk [20]. It should be noted, however, that genetic susceptibility alone is insufficient to cause T1DM, as evidenced by concordance rates of only 25–70% in monozygotic twins [21,22].

On the immunological side, autoreactive CD4+ and CD8+ T cells play a central role in insulitis, with contributions from B cells and innate immune cells, culminating in progressive β-cell destruction. Studies have indicated that the relative abundance of CD20-expressing B cells and CD28-expressing T cells may be associated with age at onset, with higher concentrations linked to earlier disease presentation [23]. On this basis, T1DM has been classified into two putative endotypes: TIDE1 (higher CD20 and CD28 expression), associated with onset in infancy, and TIDE2 (lower expression), associated with onset after age 6 [24]. These endotype classifications, however, are derived from relatively limited datasets and require independent validation before they can be used to guide clinical stratification.

Single-cell transcriptomic studies have begun to characterize the diversity of T-cell repertoires and tissue-resident immune signatures within islets, which may partly account for variability in disease progression [25]. Islet autoantibodies targeting glutamic acid decarboxylase (GAD), insulin, and tyrosine phosphatase proteins (IA-2/IA-2β) can be detectable months to years before the onset of hyperglycemia [26]. The presence of multiple autoantibodies is associated with a substantially higher likelihood of progression from the asymptomatic to the symptomatic stage [27,28]. Data from large longitudinal cohorts, including the Diabetes Autoimmunity Study in the Young (DAISY) and The Environmental Determinants of Diabetes in the Young (TEDDY), indicate that insulin autoantibodies (IAAs) typically emerge earliest, often in early childhood. Thereafter, GAD autoantibodies (GADA), IA-2, and ZnT8 autoantibodies appear [29,30]. The presence of more than two autoantibodies is associated with a markedly increased risk of progression to clinical T1DM [31].

## 4. β-Cell Fragility and “Stress” Responses

Type 1 diabetes was traditionally understood as an immune-mediated disease in which autoreactive lymphocytes progressively destroy insulin-producing β-cells [15]. Emerging evidence suggests that β-cells may not be entirely passive targets of this process, but may possess intrinsic vulnerabilities that render them susceptible to immune-mediated and metabolic injury [32]. It should be emphasized that much of this evidence is derived from in vitro studies and murine models, and its direct applicability to human T1DM remains to be established. Oxidative damage has been proposed as a contributor to β-cell dysfunction. Circulating biomarkers of oxidative damage, including malondialdehyde and protein carbonyls, have been reported to be elevated at early stages of T1DM [33,34]. β-Cells appear to be relatively susceptible to oxidative damage, partly attributed to the comparatively low expression of antioxidant enzymes such as superoxide dismutase, glutathione peroxidase, and catalase [33,35,36]. However, whether these observations reflect a primary pathogenic mechanism or a consequence of autoimmune inflammation remains unclear.

**Figure 1:**
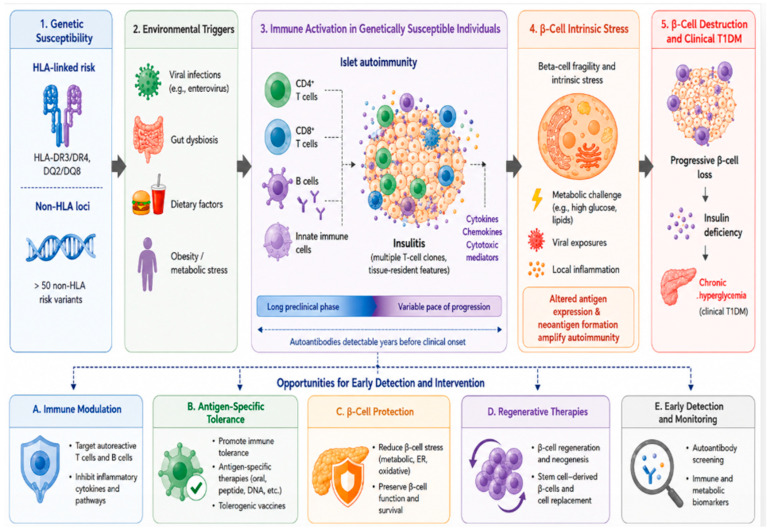
Type 1 diabetes (T1DM) multifactorial pathogenesis and opportunities for intervention.

Loss of β-cell identity has emerged as a potential additional mechanism underlying β-cell dysfunction in diabetes mellitus. Evidence from lineage-tracing studies in murine models, supported by limited human data, suggests that β-cells may undergo dedifferentiation. This process is characterized by diminished expression of critical β-cell transcription factors, including MAF bZIP transcription factor A (MAFA), and of genes required for glucose-stimulated insulin secretion, such as the glucose transporter *Slc2a2* (GLUT2) [37,38,39]. An increased prevalence of hormone-negative endocrine cells that retain chromogranin A expression has been reported in individuals with T1DM, lending some support to this concept [40]. These findings remain preliminary and cannot yet be considered as a well-established mechanism in human disease. Alterations of the endoplasmic reticulum (ER), arising from the accumulation of misfolded proteins and disturbances in calcium homeostasis, have also been implicated in β-cell dysfunction [41,42]. Persistent ER stress can trigger the unfolded protein response (UPR). When prolonged or dysregulated, UPR may activate apoptotic pathways, potentially contributing to β-cell loss [43,44]. Mitochondrial dysfunction, driven in part by autoimmune-mediated inflammation, may impair both β-cell survival and glucose-stimulated insulin secretion (GSIS) through disrupted calcium signaling and increased oxidative stress [43,45,46]. However, the precise contribution of these intracellular mechanisms to the overall rate of β-cell loss in human T1DM remains unclear. Taken together, these findings support a model in which immune dysregulation and intrinsic β-cell vulnerability may act in concert to drive disease progression. Nevertheless, the relative contribution of each suggested pathogenic process remains an area of active investigation (see Figure 1).

## 5. Environmental Triggers and Modifiers

The rising incidence of T1DM in genetically stable populations suggests that environmental factors may modify disease risk or accelerate progression in susceptible individuals [47]. Although numerous agents have been studied, including viral infections, gut microbiota, and nutritional exposures, a definitive environmental trigger has not been identified, and causal relationships remain to be established for most putative factors (see Table 1) [18].

### 5.1. Viral Infections

Epidemiological associations have been reported between various viral infections and T1DM onset, including enteroviruses, mumps, rubella, and SARS-CoV-2 [48]. It is generally considered more likely that viral infections accelerate rather than initiate the autoimmune process, potentially by increasing metabolic demand during acute illness [49]. This is because β-cell autoimmunity typically precedes clinical presentation by months to years. However, evidence for a causal role of enteroviruses remains inconsistent (see Table 1). Serological and autopsy studies show statistical associations, but causation has not been established [18].

Among the enteroviruses, Coxsackie B viruses have received the most attention. Viral RNA has been detected in pancreatic tissues, and experimental data suggest that infection may reduce NK cell activation, potentially facilitating persistent immune activation [50,51,52]. A study within the DAISY cohort reported an association between circulating Coxsackie RNA and subsequent development of islet autoantibodies in genetically susceptible children [53]. Sequence homologies between viral proteins and β-cell antigens, such as GAD, have been proposed as a mechanism for molecular mimicry, though direct experimental evidence in humans is limited [54]. Rotavirus infection before six months of age has been associated with an increased risk of IAA development in some cohort studies (see Table 1), and experimental data suggest that rotavirus can infect pancreatic cells and activate bystander immune responses, though these findings require replication [55,56,57].

For mumps, the upregulation of HLA expression on β-cells has been proposed as a mechanism to increase vulnerability to immune attack, though supporting human data are limited. Regarding SARS-CoV-2, evidence of pancreatic cell infection has been reported, and some epidemiological data suggest an increase in T1DM incidence during the COVID-19 pandemic [58,59]. Recent evidence suggests that molecular/antigen mimicry may contribute to autoimmune responses following SARS-CoV-2 infection. Churilov et al. demonstrated that SARS-CoV-2 shares peptide homology with several human endocrine autoantigens, including proteins associated with pancreatic β-cells and islet autoimmunity [60]. The study identified shared pentapeptide sequences between viral proteins and endocrine cell antigens. These findings may support the hypothesis that immune responses directed against SARS-CoV-2 may cross-react with self-antigens in genetically susceptible individuals. This mechanism has been proposed as a possible contributor to post-COVID endocrine autoimmunity, including β-cell dysfunction and the development of T1DM. Furthermore, broader reviews of coronavirus-induced autoimmunity have highlighted molecular mimicry as a central mechanism linking SARS-CoV-2 infection to autoimmune phenomena [60]. However, findings across studies have been mixed, and whether this reflects a true causal relationship, ascertainment bias, or pandemic-related changes in healthcare-seeking behavior remains uncertain. For genetically susceptible individuals, SARS-CoV-2 represents a plausible but unconfirmed candidate trigger.

### 5.2. Obesity

The relationship between obesity and T1DM is complex. While obesity is an established driver of insulin resistance in type 2 diabetes (T2DM), its role in T1DM is less clearly defined, and evidence of a direct causal relationship is limited [61]. Some studies suggest a bidirectional association between obesity and the development of T1DM. T1DM may promote weight gain through exogenous insulin administration. In addition, carbohydrate overconsumption driven by hypoglycemia avoidance may lead to obesity. The excess adiposity may, in turn, be associated with accelerated autoimmune progression in susceptible individuals [61].

A meta-analysis of 24 studies reported higher fat mass and body mass index in children with T1DM than in controls [62]. Two Polish studies reported conflicting findings: one retrospective study found no association between T1DM onset and obesity, whereas a subsequent study identified high BMI as a potential risk factor for faster β-cell depletion and increased serum levels of pro-inflammatory cytokines [63]. In adults with latent autoimmune diabetes, high BMI has been correlated with a higher frequency of IA-2 autoantibodies [64]. Maternal obesity has been associated with increased T1DM risk in offspring in some studies, though the mechanistic basis for this association remains poorly understood [61]. Preclinical studies using high-fat, high-carbohydrate diets suggest that glucotoxicity and lipotoxicity may contribute to β-cell fragility, and elevated free fatty acids may promote inflammatory signaling [61,65,66]. While these mechanistic hypotheses are biologically plausible, they are largely based on animal data, and their direct relevance to human T1DM pathogenesis has not been established (see Table 1).

### 5.3. Nutrition and Gut Health

Observational studies have examined associations between dietary patterns and T1DM risk. However, causal inferences are limited by confounding and heterogeneity across studies. Breastfeeding has been associated with a modest protective effect, while early weaning has been associated with increased risk in some cohorts [67]. Clinical trials comparing early versus late gluten introduction have not demonstrated a protective effect of delayed exposure [68]. Early introduction of cow’s milk has been associated with increased risk in epidemiological studies. It has been hypothesized that peptides in bovine proteins may resemble islet antigens, potentially stimulating cross-reactive immune responses. Amarasekara et al. (2026) reported that in healthy neonates and infants up to 4 months of age, short peptides are absorbable from the intestines, and in premature babies, may persist for a longer period of infancy due to the intestinal window period [69]. Experimental data in mice fed B1 casein support the biological plausibility of this mechanism [70], though human evidence remains associative.

Vitamin D deficiency has been associated with increased risk of T1DM, potentially reflecting its immunomodulatory functions (see Table 1). In vitro studies have demonstrated that vitamin D can protect β-cells from inflammation-induced exhaustion, in part by activating BRG1/BRM-associated factor (BAF) chromatin-remodeling complexes [71]. A recent clinical trial reported that vitamin D supplementation reduced the plasma TNF-α levels and insulin requirements [72]. However, this is a single trial, and its findings require replication before firm conclusions can be drawn.

Intestinal permeability has been proposed as a contributor to T1DM pathogenesis. Cross-sectional and prospective studies suggest that increased intestinal permeability may precede the symptomatic stage of T1DM [73,74]. A study by Harbison et al. (2019) reported that children with islet autoimmunity exhibited gut dysbiosis, characterized by reductions in *Prevotella* and *Butyricimonas* genera and lower abundance of short-chain fatty acid (SCFA)-producing bacteria, and that those who progressed to clinical T1DM had greater intestinal permeability than non-progressors [74]. An experimental murine study by Pöystiet al. (2023) demonstrated induced gut dysbiosis. These findings were associated with increased intestinal permeability, elevated circulating endotoxin levels, and promoted CXCL10 production by islet macrophages and β-cells [75]. Zonulin, a protein that regulates tight junction permeability, has been found to be elevated prior to the symptomatic stage of T1DM in some studies [76,77]. These findings are consistent with a role for gut barrier dysfunction in T1DM pathogenesis, though the available evidence is largely observational or derived from animal models. Whether increased intestinal permeability is a cause, a consequence, or an epiphenomenon of the autoimmune process remains unclear.

## 6. Opportunities for Early Detection and Intervention

Informed by the pathophysiological framework outlined above, several preventive strategies have been investigated that target distinct stages of the disease process. These include immunomodulatory therapies, antigen-specific tolerance induction, antioxidant approaches, vaccine strategies, and regenerative medicine. The evidence base varies considerably across these areas, and the section below summarizes the current findings with attention to the level of evidence supporting each approach.

### 6.1. Advances in Early Detection

Identification of high-risk individuals before clinical onset is central to prevention strategies. Contemporary screening programs integrate genetic risk scoring with islet autoantibody testing, enabling classification into defined disease stages, i.e., autoimmunity without dysglycemia (Stage 1), dysglycemia without clinical diabetes (Stage 2), and symptomatic disease (Stage 3) [12,78]. Early detection programs in Europe, the United States, and Australia have demonstrated reductions in diabetic ketoacidosis (DKA) at diagnosis and facilitated enrolment into prevention trials, though population-level impact data remain limited [79,80,81]. Screening is currently largely directed at first-degree relatives of affected individuals, who carry a 15–20-fold higher risk than the general population [82,83]. Concordance rates in monozygotic twins of 25–70%, compared with 6–7% in dizygotic twins and siblings, highlight the contribution of both genetic and non-genetic factors [21,22]. In a retrospective cohort of 3015 first-degree relatives, 1.59% were islet autoantibody-positive, and more than half progressed to T1DM within five years [81]. Structured screening initiatives such as TrialNet and the Diabetes Prevention Trial (DPT-1) have built on these findings [22,84,85]. The DPT-1 screened over 80,000 individuals and evaluated whether parenteral or oral insulin could delay T1DM in high-risk relatives; however, neither intervention reduced diabetes incidence significantly [85,86]. These findings highlight the difficulty of translating pathophysiological hypotheses into effective preventive interventions.

Individuals with other autoimmune conditions, particularly autoimmune thyroid disease and coeliac disease, are at increased risk of T1DM, reflecting shared immunogenetic susceptibility [21,87]. Data from the Autoimmunity Screening for Kids (ASK) study in Colorado demonstrated a higher prevalence of multiple islet autoantibodies in children with coeliac disease or a family history of coeliac disease [88]. Autoantibody detection in this program used high-affinity radiobinding assays (RBAs) and electrochemiluminescence (ECL) techniques for GAD, IAA, IA-2A, and ZnT8. The persistent positivity triggered follow-up at 3–6-month intervals, with oral glucose tolerance testing (OGTT) and optional continuous glucose monitoring (CGM) for high-risk subgroups [89,90].

Several important limitations complicate the early detection of T1DM. A minority of children with T1DM are antibody-negative at diagnosis, potentially representing true idiopathic (type 1B) diabetes, testing performed before seroconversion, or misclassification of monogenic forms [90,91,92]. Autoantibody profiles and HLA susceptibility patterns differ across ethnic groups, which may limit the sensitivity and generalizability of antibody-based diagnostic algorithms in non-European populations [93]. In many low- and middle-income settings, including parts of sub-Saharan Africa, children frequently present with advanced DKA, reflecting limited public awareness, restricted access to healthcare, and misdiagnosis as common infectious or gastrointestinal conditions [94,95].

C-peptide measurement is useful for assessing residual β-cell function but is subject to important interpretive challenges in pediatric populations. These include physiological variation with age and puberty, overlap with early T2DM, and the confounding effect of the transient post-diagnosis “honeymoon phase”. During this phase, partial β-cell recovery may occur, leading to a temporary decrease in insulin requirements [96,97,98]. In adults, distinguishing T1DM from T2DM remains challenging, as the conditions share overlapping clinical features. Therefore, misclassification becomes more common with increasing age at onset [99,100,101]. Approximately one in six individuals with a clinical diagnosis of T1DM may retain C-peptide levels within the T2DM range and have low autoantibody positivity [99,100].

Conversely, a substantial proportion of individuals who ultimately develop severe insulin deficiency consistent with T1DM are not commenced on insulin at diagnosis [102,103]. Genetic risk analyses from large UK cohorts suggest that approximately two-thirds of adults who are autoantibody-negative but treated as having T1DM are unlikely to have true autoimmune T1DM [101]. These diagnostic uncertainties highlight the need for improved biomarkers and classification criteria, particularly for adult-onset disease (see Table 1).

### 6.2. Immunomodulatory Prevention Strategies

Immunomodulatory therapies aim to attenuate the autoimmune destruction of β-cells. Teplizumab, an anti-CD3 monoclonal antibody, has demonstrated the most significant clinical advance to date (see Table 1). This biologic has achieved regulatory approval based on a phase 2 trial that showed it delayed progression from Stage 2 to Stage 3 T1DM by a median of approximately 48 months in approximately 43% of participants [104,105,106]. This represents the first disease-modifying therapy approved for the prevention of T1DM. However, important limitations should be noted. The therapeutic responses were heterogeneous, systemic administration was associated with a high frequency of adverse events (at least one event in 99.5% of participants across five clinical trials, including lymphopenia, which was generally transient) [107,108], and the long-term durability of the effect remains uncertain.

A further challenge is the apparent dissociation between biomarker and clinical outcomes observed across immunotherapy trials (see Table 1). Meta-analyses of non-antigen-specific immunotherapies consistently demonstrate the preservation of C-peptide and reductions in exogenous insulin requirements, yet these effects have generally not been accompanied by statistically significant improvements in HbA1c or fasting plasma glucose [109]. The Protégé trial, for example, reported significant C-peptide preservation at two years with teplizumab but no significant difference in HbA1c between the treatment and placebo groups at any time point [110]. Whether this discordance reflects limitations in trial design, insufficient statistical power, or genuine biological constraints on the extent to which β-cell preservation translates into glycemic improvement remains contested [111]. Resolving this question has important implications for trial endpoint selection and future regulatory pathways.

Other immune-targeted approaches under evaluation include rituximab (anti-CD20). The administration of rituximab results in the depletion of autoantibody-producing B cells and delayed C-peptide decline by approximately 8.2 months in a phase 2 trial of recent-onset T1DM [112]. A subsequent trial combining rituximab with autologous expanded regulatory T cells (Tregs) did not achieve statistically significant delay in disease progression, despite numerically improved outcomes compared with Treg monotherapy [113]. Adverse events were reported in 93% and 80% of participants in these two trials, respectively [83,113]. Abatacept (costimulation blockade), low-dose IL-2 (to expand regulatory T cells), and golimumab (anti-TNF-α) have each shown biological activity in phase 2 trials, including a 43% reduction in C-peptide decline with golimumab after 52 weeks [114,115]. However, none has yet demonstrated durable, clinically meaningful endpoints, and the overall evidence base for these agents remains insufficient to support routine use outside of clinical trials.

Cytokine-blockade strategies have demonstrated limited efficacy. A phase 2 trial of tocilizumab (IL-6 receptor inhibitor) did not prevent β-cell functional decline or alter T-cell frequencies [116]. Similarly, a phase 2 trial of ladarixin (IL-8 receptor inhibitor) failed to demonstrate efficacy in patients with newly diagnosed disease [117]. Both studies reported acceptable tolerability (see Table 1).

### 6.3. Vaccine Strategies

Viral associations with T1DM have motivated vaccine-based prevention strategies. Observational data have suggested a reduced incidence of T1DM in rotavirus-vaccinated cohorts. A large nationwide cohort study by Rogers et al. (2019) reported a 33% reduction in T1DM risk associated with the completion of all rotavirus vaccine doses [118]. This finding has been partially corroborated by a subsequent meta-analysis reporting a 13% risk reduction [119]. Whilst these findings are promising, they are observational findings, and may be subject to confounding; randomized trial data are not available (see Table 1).

The Bacillus Calmette–Guérin (BCG) vaccine has been proposed to confer some protection against T1DM progression, with one study by Doupis et al. (2021) reporting a delay of approximately 2.5 years with a single dose administered at age 9 [120]. The proposed mechanism involves the immunomodulation of T-lymphocyte responses, favoring regulatory and cytotoxic T-cell populations. However, this evidence is limited to a small number of studies, and the finding requires replication in adequately powered trials.

A multivalent inactivated Coxsackie B vaccine was evaluated in a phase 1 trial in adults (NCT04690426) [121]. The vaccine was immunogenic and well-tolerated, with no evidence of T1DM induction or an increase in islet autoantibodies in genetically susceptible participants [121]. Whether it confers protection against T1DM development has not yet been established and requires further investigation.

### 6.4. Antioxidant Strategies

Given evidence implicating oxidative damage in β-cell vulnerability, antioxidant supplementation has been investigated as an adjunctive strategy (see Table 1). Vitamin E, a lipid-soluble antioxidant, has been the most extensively studied agent in T1DM. A clinical study by Gupta et al. in 40 children (20 with T1DM, 20 controls) demonstrated that 600 mg of vitamin E daily for 3 months significantly reduced the malondialdehyde (MDA) levels and increased glutathione (GSH) concentrations in the T1DM group, which suggests enhanced antioxidant defense [122]. A subsequent meta-analysis of 7 randomized controlled trials reported that vitamin E supplementation was well-tolerated and associated with a significantly lower HbA1c than the placebo [123]. These findings are promising; however, the trials included are small, heterogeneous, and of variable quality, and larger confirmatory studies are needed before vitamin E supplementation can be recommended as a preventive strategy (see Table 1).

### 6.5. Antigen-Specific and Tolerogenic Therapies

Antigen-specific approaches seek to re-establish immune tolerance to β-cell antigens without broadly suppressing the immune system. Clinical trials have evaluated oral and nasal insulin administration, peptide-based immunotherapy, and tolerogenic dendritic cell strategies [124,125,126,127]. To date, none of these approaches has demonstrated definitive efficacy in preventing or substantially delaying the onset of clinical T1DM in adequately powered trials. The evidence remains at an early stage, and these strategies are best regarded as promising avenues for further investigation rather than established interventions (see Table 1).

### 6.6. Regenerative and β-Cell Replacement Approaches

Regenerative strategies aim to restore insulin secretory capacity rather than prevent autoimmune destruction. Stem-cell-derived β-like cells and encapsulated islet transplantation have entered early-phase clinical trials, with some reports of insulin independence in selected recipients [128,129,130]. Gene-editing technologies have been explored to develop immune-evasive β-cells that resist rejection and autoimmune attack [131]. While these advances represent an important frontier in the field, major challenges remain regarding scalability, the long-term durability of transplanted cells, and the adequacy of immune protection strategies. Current evidence is insufficient to evaluate the clinical utility of these approaches, and they should be considered investigational (see Table 1).

## 7. Authors’ Perspective and Conclusions

Advances in understanding T1DM pathophysiology have identified a window of opportunity for early intervention. The demonstration that islet autoantibodies emerge months to years before clinical onset, and that the disease can be staged on the basis of autoimmunity and glycemic status, has transformed prevention from a theoretical goal to an active area of clinical investigation. The regulatory approval of teplizumab, which delays progression from Stage 2 to Stage 3 T1DM by a median of approximately 4 years, represents the most important clinical milestone to date, establishing proof-of-concept for a disease-modifying therapy [104,105,106]. Nevertheless, it is important to emphasize that teplizumab remains the exception rather than the rule, i.e., the vast majority of preventive strategies investigated to date remain investigational, and none beyond selected immunomodulatory therapies has demonstrated sufficient efficacy in adequately powered trials to support routine clinical use.

Several evidence-based priorities emerge from current knowledge. First, broader implementation of genetic risk scoring and autoantibody screening may facilitate the earlier identification of individuals at increased risk, particularly among first-degree relatives of affected individuals, who carry a 15–20-fold higher risk than the general population. In most settings, autoantibody testing is currently reserved for diagnostic confirmation. Given that autoimmunity precedes clinical onset by years, earlier deployment as a risk-stratification tool is scientifically justified and increasingly feasible. Development of affordable, scalable diagnostic platforms will be essential to extend these benefits to low- and middle-income settings.

Second, viral triggers, particularly enteroviruses, remain a compelling but unconfirmed target for vaccine-based prevention. The phase 1 trial of the Coxsackie B vaccine PRV-101 demonstrated acceptable safety and immunogenicity. However, whether it confers meaningful protection against T1DM remains to be evaluated in adequately powered phase 2 and phase 3 trials [120]. Future studies will need to carefully evaluate the potential implications of molecular mimicry when developing vaccine-based prevention strategies, given the theoretical risk that immune responses directed against viral antigens could cross-react with β-cell autoantigens in genetically susceptible individuals.

Third, despite biological plausibility, the evidence linking early nutritional exposures, antioxidant supplementation, and gut barrier function to T1DM risk remains largely observational or preclinical. These strategies should currently be regarded as hypothesis-generating rather than practice-changing. Prospective intervention studies are needed to determine whether modifiable dietary factors, microbiome-targeted strategies, or antioxidant approaches can meaningfully reduce disease incidence in at-risk populations before any clinical recommendations can be made.

Important unresolved questions continue to limit progress. The mechanistic basis for the dissociation between C-peptide preservation and glycemic improvement observed across immunotherapy trials remains unclear, and its resolution is critical for future trial design and endpoint selection. The relative contributions of immune dysregulation and intrinsic β-cell vulnerability to disease progression remain incompletely understood. Recruitment of children into prevention trials is complicated by the early age of onset, and the anatomical inaccessibility of the pancreas constrains direct mechanistic study in humans. Finally, the multifactorial etiology of T1DM suggests that no single intervention is likely to be sufficient; combination strategies targeting both immune and β-cell pathways will probably be necessary for durable disease modification.

In summary, while the field has advanced substantially from purely reactive management toward stage-specific prevention, this progress must be interpreted with appropriate caution. Autoantibody-based staging and teplizumab represent genuinely established advances; however, regenerative approaches, antigen-specific tolerogenic therapies, vaccine strategies, microbiota-directed interventions, and antioxidant supplementation remain at early or investigational stages, and their clinical utility has not yet been established. Translating current promise into population-level benefit will require scalable screening infrastructure, rigorous replication of early-phase findings in larger and more diverse trials, and a clearer understanding of which biological pathways are most amenable to intervention at each stage of disease.

## Figures and Tables

**Table 1:** Summary of current and emerging strategies for the early detection and prevention of type 1 diabetes (T1DM).

Strategy	Proposed Mechanism	Level of Evidence	Current Clinical Readiness	Major Limitation
Autoantibody-based staging (GAD, IAA, IA-2A, ZnT8) + genetic risk scoring	Identification of individuals at increased risk and classification into Stages 1–3 disease before clinical onset	Strong evidence from prospective cohort studies. Established staging framework (Stages 1–3).	Established and implemented in research and selected clinical screening programs (TrialNet, ASK, Fr1da).	Limited accessibility in some regions (low- and middle-income countries. Autoantibody profiles vary across ethnic groups (lower sensitivity in non-European). Some individuals remain autoantibody-negative.
Teplizumab (anti-CD3 monoclonal antibody)	Delays progression from Stage 2 to Stage 3 T1DM through modulation of autoreactive T-cell responses.	Strong evidence from phase 2 clinical trials. Regulatory approval granted.	Approved for delaying progression to clinical T1DM in high-risk individuals. First disease-modifying therapy.	Variable treatment response. Frequent adverse events. Long-term durability remains uncertain.
Other immunomodulatory therapies (rituximab, abatacept, low-dose IL-2, golimumab)	Preservation of residual β-cell function through modulation of immune pathways.	Moderate evidence from phase 2 clinical trials.	Investigational.	Limited evidence for durable clinical benefit. Adverse events common. Not approved for routine prevention.
Cytokine-targeted therapies (tocilizumab, ladarixin)	Inhibition of pro-inflammatory cytokine signaling.	Limited evidence from phase 2 trials.	Investigational.	Trials have not demonstrated meaningful efficacy despite acceptable tolerability.
Rotavirus vaccination	Potential reduction of virus-associated autoimmune triggers.	Observational evidence and meta-analytic support.	Established as a public health vaccine, but not specifically for T1DM prevention.	Evidence is observational. Causality remains unproven. No randomized prevention trials.
BCG vaccination	Proposed immunomodulation favoring regulatory immune responses.	Limited evidence from a small number of studies.	Investigational for T1DM prevention.	Requires replication in larger, adequately powered trials.
Coxsackie B virus vaccine	Prevention of enterovirus-associated autoimmune activation.	Early evidence from the phase 1 clinical trial (adults).	Early-stage clinical development.	Immunogenicity and safety demonstrated. Protective efficacy against T1DM has not yet been demonstrated.
Antioxidant supplementation (vitamin E)	Reduction of oxidative stress and enhancement of antioxidant defense mechanisms.	Limited-to-moderate evidence from small clinical studies and meta-analysis.	Investigational.	Small and heterogeneous studies. Insufficient evidence for routine preventive use.
Microbiota-directed interventions (targeting gut dysbiosis, intestinal permeability)	Modulation of gut microbiota and restoration of intestinal barrier integrity to reduce immune dysregulation, inflammatory signaling, and β-cell autoimmunity.	Observational and animal studies.	Pre-clinical/early investigational.	Evidence largely observational or murine; causality not established; no clinical trial data reported in this review.
Antigen-specific and tolerogenic therapies (oral/nasal insulin, peptide immunotherapy, tolerogenic dendritic cells)	Restoration of immune tolerance to β-cell antigens without broad immunosuppression.	Limited evidence from clinical trials.	Investigational.	No definitive efficacy demonstrated in adequately powered studies.
Regenerative and β-cell replacement approaches (stem-cell-derived β-cells, encapsulated islets, gene-edited β-cells)	Restoration of insulin-producing β-cell mass.	Early clinical and preclinical evidence.	Experimental.	Challenges related to scalability, immune protection, long-term durability, and cost.

## Data Availability

No new data were created or analyzed in this study.

## Abbreviations

The following abbreviations are used in this manuscript:

GAD	Glutamic Acid Decarboxylase
BCG	Bacillus Calmette–Guérin
ECL	Electrochemiluminescence
DAISY	Diabetes Autoimmunity Study in the Young
DKA	Diabetic Ketoacidosis
HLA	Human Leukocyte Antigen
UPR	Unfolded Protein Response
GLUT2	Glucose transporter 2
ROS	Reactive Oxygen Species
CGM	Continuous Glucose Monitoring
MCU	Mitochondrial Calcium Uniporter
GSH	Glutathione
MDA	Malondialdehyde
T1DM	Type 1 Diabetes Mellitus
T2DM	Type 2 Diabetes Mellitus
TEDDY	The Environmental Determinants of Diabetes in the Young
SCFA	Short-Chain Fatty Acid
BAF	BRG1/BRM-Associated Factor
IAAs	Insulin Autoantibodies
GADAs	Glutamic Acid Decarboxylase Antibodies
MAFA	MAF bZIP Transcription Factor A
ASK	Autoimmunity Screening for Kids

## References

[1] Addissouky T.A., Ali M.M.A., El Sayed I.E.T., Wang Y. (2024). Type 1 diabetes mellitus: Retrospect and prospect. Bull. Natl. Res. Cent..

[2] Ilonen J., Lempainen J., Veijola R. (2019). The heterogeneous pathogenesis of type 1 diabetes melli-tus. Nat. Rev. Endocrinol..

[3] von Scholten B.J., Kreiner F.F., Gough S.C.L., von Herrath M. (2021). Current and future therapies for type 1 diabetes. Diabetologia.

[4] Noble J.A., Henry A.E. (2012). Genetics of type 1 diabetes. Cold Spring Harb. Perspect. Med..

[5] Mauvais F.X., van Endert P.M. (2025). Type 1 Diabetes: A Guide to Autoimmune Mechanisms for Clinicians. Diabetes Obes. Metab..

[6] Yau C., Danska J.S. (2024). Cracking the type 1 diabetes code: Genes, microbes, immunity, and the early life environment. Immunol. Rev..

[7] Liu B., Shao Y., Fu R. (2021). Current research status of HLA in immune-related diseases. Immun. Inflamm. Dis..

[8] Crux N.B., Elahi S. (2017). Human Leukocyte Antigen (HLA) and Immune Regulation: How Do Classical and Non-Classical HLA Alleles Modulate Immune Response to Human Immunodeficiency Virus and Hepatitis C Virus Infections?. Front. Immunol..

[9] Lernmark Å. (2021). Etiology of Autoimmune Islet Disease: Timing Is Everything. Diabetes.

[10] La Noce M., Nicoletti G.F., Papaccio G., Del Vecchio V., Papaccio F. (2022). Insulitis in Human Type 1 Diabetic Pancreas: From Stem Cell Grafting to Islet Organoids for a Successful Cell-Based Therapy. Cells.

[11] Walker S.L., Leete P., Boldison J. (2025). Tissue Resident and Infiltrating Immune Cells: Their Influence on the Demise of Beta Cells in Type 1 Diabetes. Biomolecules.

[12] Leichter S.B., Felton J.L., Geno Rasmussen C., Rizzuto P., Bellini N., Ebekozien O., Schulman-Rosenbaum R. (2025). Establishing Screening Programs for Presymptomatic Type 1 Diabetes: Practical Guidance for Diabetes Care Providers. J. Clin. Endocrinol. Metab..

[13] Mallone R., Halliez C., Rui J., Herold K.C. (2022). The β-Cell in Type 1 Diabetes Pathogenesis: A Victim of Circumstances or an Instigator of Tragic Events?. Diabetes.

[14] Kulkarni A., Muralidharan C., May S.C., Tersey S.A., Mirmira R.G. (2022). Inside the β Cell: Molecular Stress Response Pathways in Diabetes Pathogenesis. Endocrinology.

[15] Toren E., Burnette K.S., Banerjee R.R., Hunter C.S., Tse H.M. (2021). Partners in Crime: Beta-Cells and Autoimmune Responses Complicit in Type 1 Diabetes Pathogenesis. Front. Immunol..

[16] Laine A.P., Valta M., Toppari J., Knip M., Veijola R., Ilonen J., Lempainen J. (2022). Non-HLA Gene Polymorphisms in the Pathogenesis of Type 1 Diabetes: Phase and Endotype Specific Effects. Front. Immunol..

[17] Pociot F., Lernmark Å. (2016). Genetic risk factors for type 1 diabetes. Lancet.

[18] Aamodt K.I., Powers A.C. (2025). The pathophysiology, presentation and classification of Type 1 diabetes. Diabetes Obes. Metab..

[19] Erlich H., Valdes A.M., Noble J., Carlson J.A., Varney M., Concannon P., Mychaleckyj J.C., Todd J.A., Bonella P., Fear A.L. (2008). Type 1 Diabetes Genetics Consortium. HLA DR-DQ haplotypes and genotypes and type 1 diabetes risk: Analysis of the type 1 diabetes genetics consortium families. Diabetes.

[20] Bjørnvold M., Undlien D.E., Joner G., Dahl-Jørgensen K., Njølstad P.R., Akselsen H.E., Gervin K., Rønningen K.S., Stene L.C. (2008). Joint effects of HLA, INS, PTPN22 and CTLA4 genes on the risk of type 1 diabetes. Diabetologia.

[21] Flores Monar G.V., Islam H., Puttagunta S.M., Islam R., Kundu S., Jha S.B., Rivera A.P., Sange I. (2022). Association Between Type 1 Diabetes Mellitus and Celiac Disease: Autoimmune Disorders with a Shared Genetic Background. Cureus.

[22] Primavera M., Giannini C., Chiarelli F. (2020). Prediction and Prevention of Type 1 Diabetes. Front. Endocrinol..

[23] Wang Y.N., Li R., Huang Y., Chen H., Nie H., Liu L., Zou X., Zhong J., Zheng B., Gong Q. (2024). The role of B cells in the pathogenesis of type 1 diabetes. Front. Immunol..

[24] Battaglia M., Ahmed S., Anderson M.S., Atkinson M.A., Becker D., Bingley P.J., Bosi E., Brusko T.M., DiMeglio L.A., Evans-Molina C. (2020). Introducing the Endotype Concept to Address the Challenge of Disease Heterogeneity in Type 1 Diabetes. Diabetes Care.

[25] Jung S., Lee J.S. (2023). Single-Cell Genomics for Investigating Pathogenesis of Inflammatory Diseases. Mol. Cells.

[26] Kawasaki E. (2023). Anti-Islet Autoantibodies in Type 1 Diabetes. Int. J. Mol. Sci..

[27] So M., Speake C., Steck A.K., Lundgren M., Colman P.G., Palmer J.P., Herold K.C., Greenbaum C.J. (2021). Advances in type 1 diabetes prediction using islet autoantibodies: Beyond a simple count. Endocr. Rev..

[28] Jia X., Yu L. (2021). Understanding islet autoantibodies in prediction of type 1 diabetes. J. Endocr. Soc..

[29] Stanley H.M., Norris J.M., Barriga K., Hoffman M., Yu L., Miao D., Erlich H.A., Eisenbarth G.S., Rewers M. (2004). Is presence of islet autoantibodies at birth associated with development of persistent islet autoimmunity? The Diabetes Autoimmunity Study in the Young (DAISY). Diabetes Care.

[30] Frohnert B.I., Ide L., Dong F., Barón A.E., Steck A.K., Norris J.M., Rewers M.J. (2017). Late-onset islet autoimmunity in childhood: The Diabetes Autoimmunity Study in the Young (DAISY). Diabetologia.

[31] Jacobsen L.M., Bocchino L., Evans-Molina C., DiMeglio L., Goland R., Wilson D.M., Atkinson M.A., Aye T., Russell W.E., Wentworth J.M. (2020). The risk of progression to type 1 diabetes is highly variable in individuals with multiple autoantibodies following screening. Diabetologia.

[32] James E.A., Joglekar A.V., Linnemann A.K., Russ H.A., Kent S.C. (2023). The beta cell-immune cell interface in type 1 diabetes (T1D). Mol. Metab..

[33] Leenders F., Groen N., de Graaf N., Engelse M.A., Rabelink T.J., de Koning E.J.P., Carlotti F. (2021). Oxidative Stress Leads to β-Cell Dysfunction Through Loss of β-Cell Identity. Front. Immunol..

[34] Dominguez C., Ruiz E., Gussinye M., Carrascosa A. (1998). Oxidative Stress at Onset and in Early Stages of Type 1 Diabetes in Children and Adolescents. Diabetes Care.

[35] Lortz S., Tiedge M., Lenzen S. (1997). Relation Between Antioxidant Enzyme Gene Expression and Antioxidative Defense Status of Insulin-Producing Cells. Implications from Studies on Bioengineered RINm5F Cells. Exp. Clin. Endocrinol. Diabetes.

[36] Tonooka N., Oseid E., Zhou H., Harmon J.S., Robertson R.P. (2007). Glutathione Peroxidase Protein Expression and Activity in Human Islets Isolated for Transplantation. Clin. Transplant..

[37] Talchai C., Xuan S., Lin H.V., Sussel L., Accili D. (2012). Pancreatic β Cell Dedifferentiation as a Mechanism of Diabetic β Cell Failure. Cell.

[38] Accili D., Talchai S.C., Kim-Muller J.Y., Cinti F., Ishida E., Ordelheide A.M., Kuo T., Fan J., Son J. (2016). When β-Cells Fail: Lessons from Dedifferentiation. Diabetes Obes. Metab..

[39] Swisa A., Glaser B., Dor Y. (2017). Metabolic Stress and Compromised Identity of Pancreatic Beta Cells. Front. Genet..

[40] Moin A.S.M., Dhawan S., Shieh C., Butler P.C., Cory M., Butler A.E. (2016). Increased Hormone-Negative Endocrine Cells in the Pancreas in Type 1 Diabetes. J. Clin. Endocrinol. Metab..

[41] Zhang Z.W., Cheng J., Xu F., Chen Y.E., Du J.B., Yuan M., Zhu F., Xu X.C., Yuan S. (2011). Red blood cell extrudes nucleus and mitochondria against oxidative stress. IUBMB Life.

[42] Bogenhagen D.F. (2012). Mitochondrial DNA nucleoid structure. Biochim. Biophys. Acta.

[43] Diane A., Al-Shukri N.A., Bin Abdul Mu-U-Min R., Al-Siddiqi H.H. (2022). β-cell mitochondria in diabetes mellitus: A missing puzzle piece in the generation of hPSC-derived pancreatic β-cells?. J. Transl. Med..

[44] Giacomello M., Pyakurel A., Glytsou C., Scorrano L. (2020). The cell biology of mitochondrial membrane dynamics. Nat. Rev. Mol. Cell Biol..

[45] Matsumoto S., Shimoda M. (2020). Current situation of clinical islet transplantation from allogeneic toward xenogeneic. J. Diabetes.

[46] Maxwell K.G., Millman J.R. (2021). Applications of iPSC-derived beta cells from patients with diabetes. Cell Rep. Med..

[47] Norris J.M., Johnson R.K., Stene L.C. (2020). Type 1 diabetes-early life origins and changing epidemiology. Lancet Diabetes Endocrinol..

[48] Craig M.E., Nair S., Stein H., Rawlinson W.D. (2013). Viruses and type 1 diabetes: A new look at an old story. Pediatr. Diabetes.

[49] Dahlquist G. (2006). Can we slow the rising incidence of childhood-onset autoimmune diabetes? The overload hypothesis. Diabetologia.

[50] Tracy S., Smithee S., Alhazmi A., Chapman N. (2015). Coxsackievirus can persist in murine pancreas by deletion of 5′ terminal genomic sequences. J. Med. Virol..

[51] Alidjinou E.K., Engelmann I., Bossu J., Villenet C., Figeac M., Romond M.B., Sané F., Hober D. (2017). Persistence of Coxsackievirus B4 in pancreatic ductal-like cells results in cellular and viral changes. Virulence.

[52] Nekoua M.P., Bertin A., Sane F., Alidjinou E.K., Lobert D., Trauet J., Hober C., Engelmann I., Moutairou K., Yessoufou A. (2020). Pancreatic beta cells persistently infected with coxsackievirus B4 are targets of NK cell-mediated cytolytic activity. Cell. Mol. Life Sci..

[53] Stene L.C., Oikarinen S., Hyöty H., Barriga K.J., Norris J.M., Klingensmith G., Hutton J.C., Erlich H.A., Eisenbarth G.S., Rewers M. (2010). Enterovirus infection and progression from islet autoimmunity to type 1 diabetes: The Diabetes and Autoimmunity Study in the Young (DAISY). Diabetes.

[54] Nekoua M.P., Alidjinou E.K., Hober D. (2022). Persistent coxsackievirus B infection and pathogenesis of type 1 diabetes mellitus. Nat. Rev. Endocrinol..

[55] Lempainen J., Tauriainen S., Vaarala O., Mäkelä M., Honkanen H., Marttila J., Veijola R., Simell O., Hyöty H., Knip M. (2012). Interaction of enterovirus infection and cow’s milk-based formula nutrition in type 1 diabetes-associated autoimmunity. Diabetes/Metab. Res. Rev..

[56] Shulman L.M., Hampe C.S., Ben-Haroush A., Perepliotchikov Y., Vaziri-Sani F., Israel S., Miller K., Bin H., Kaplan B., Laron Z. (2014). Antibodies to islet cell autoantigens, rotaviruses and/or enteroviruses in cord blood and healthy mothers in relation to the 2010–2011 winter viral seasons in Israel: A pilot study. Diabet. Med..

[57] Burke R.M., Tate J.E., Jiang B., Parashar U.D. (2020). Rotavirus and Type 1 Diabetes-Is There a Connection? A Synthesis of the Evidence. J. Infect. Dis..

[58] Müller J.A., Groß R., Conzelmann C., Krüger J., Merle U., Steinhart J., Weil T., Koepke L., Bozzo C.P., Read C. (2021). SARS-CoV-2 infects and replicates in cells of the human endocrine and exocrine pancreas. Nat. Metab..

[59] Wu C.T., Lidsky P.V., Xiao Y., Lee I.T., Cheng R., Nakayama T., Jiang S., Demeter J., Bevacqua R.J., Chang C.A. (2021). SARS-CoV-2 infects human pancreatic β cells and elicits β cell impairment. Cell Metab..

[60] Churilov L.P., Normatov M.G., Utekhin V.J. (2022). Molecular Mimicry between SARS-CoV-2 and Human Endocrinocytes: A Prerequisite of Post-COVID-19 Endocrine Autoimmunity?. Pathophysiology.

[61] Kueh M.T., Chew N.W., Al-Ozairi E., le Roux C.W. (2024). The emergence of obesity in type 1 diabetes. Int. J. Obes..

[62] Zheng Y., Rostami Haji Abadi M., Gough J., Johnston J.J.D., Nour M., Kontulainen S. (2022). Higher Body Fat in Children and Adolescents with Type 1 Diabetes–A Systematic Review and Meta-Analysis. Front. Pediatr..

[63] Wasyl-Nawrot B., Wójcik M., Nazim J., Skupień J., Starzyk J.B. (2020). Increased incidence of type 1 diabetes in children and no change in the age of diagnosis and BMI-SDS at the onset-is the accelerator hypothesis not working?. J. Clin. Res. Pediatr. Endocrinol..

[64] Kurpiewska E., Ciężki S., Jamiołkowska-Sztabkowska M., Polkowska A., Starosz A., Grubczak K., Moniuszko M., Bossowski K., Głowińska-Olszewska B. (2023). Excessive BMI is associated with higher C-peptide level at recognition but also with its greater loss in two years clinical observation in children with new onset type 1 diabetes. Front. Immunol..

[65] March C.A., Becker D.J., Libman I.M. (2021). Nutrition and Obesity in the Pathogenesis of Youth-Onset Type 1 Diabetes and Its Complications. Front. Endocrinol..

[66] Corbin K.D., Driscoll K.A., Pratley R.E., Smith S.R., Maahs D.M., Mayer-Davis E.J. (2018). Advancing Care for Type 1 Diabetes and Obesity Network (ACT1ON). Obesity in Type 1 Diabetes: Pathophysiology, Clinical Impact, and Mechanisms. Endocr. Rev..

[67] Lampousi A.M., Carlsson S., Löfvenborg J.E. (2021). Dietary factors and risk of islet autoimmunity and type 1 diabetes: A systematic review and meta-analysis. eBioMedicine.

[68] Hummel S., Pflüger M., Hummel M., Bonifacio E., Ziegler A.G. (2011). Primary dietary intervention study to reduce the risk of islet autoimmunity in children at increased risk for type 1 diabetes: The BABYDIET study. Diabetes Care.

[69] Amarasekara Y., Sunna A., German J.B. (2026). From bench to crib: Translating human milk bioactive peptides into infant health interventions. Crit. Rev. Food Sci. Nutr..

[70] Chia J.S.J., McRae J.L., Enjapoori A.K., Lefèvre C.M., Kukuljan S., Dwyer K.M. (2018). Dietary Cows’ Milk Protein A1 Beta-Casein Increases the Incidence of T1D in NOD Mice. Nutrients.

[71] Wei Z., Yoshihara E., He N., Hah N., Fan W., Pinto A.F., Huddy T., Wang Y., Ross B., Estepa G. (2018). Vitamin D switches BAF complexes to protect β cells. Cell.

[72] Nwosu B.U., Parajuli S., Jasmin G., Fleshman J., Sharma R.B., Alonso L.C., Lee A.F., Barton B.A. (2021). Ergocalciferol in New-onset Type 1 Diabetes: A Randomized Controlled Trial. J. Endocr. Soc..

[73] Bosi E., Molteni L., Radaelli M.G., Folini L., Fermo I., Bazzigaluppi E., Piemonti L., Pastore M.R., Paroni R. (2006). Increased intestinal permeability precedes clinical onset of type 1 diabetes. Diabetologia.

[74] Harbison J.E., Roth-Schulze A.J., Giles L.C., Tran C.D., Ngui K.M., Penno M.A., Thomson R.L., Wentworth J.M., Colman P.G., Craig M.E. (2019). Gut microbiome dysbiosis and increased intestinal permeability in children with islet autoimmunity and type 1 diabetes: A prospective cohort study. Pediatr. Diabetes.

[75] Pöysti S., Silojärvi S., Brodnicki T.C., Catterall T., Liu X., Mackin L., Luster A.D., Kay T.W., Christen U., Thomas H.E. (2023). Gut dysbiosis promotes islet autoimmunity by increasing T-cell attraction in islets via CXCL10 chemokine. J. Autoimmun..

[76] Sapone A., De Magistris L., Pietzak M., Clemente M.G., Tripathi A., Cucca F., Lampis R., Kryszak D., Cartenì M., Generoso M. (2006). Zonulin upregulation is associated with increased gut permeability in subjects with type 1 diabetes and their relatives. Diabetes.

[77] Joesten W.C., Short A.H., Kennedy M.A. (2019). Spatial variations in gut permeability are linked to type 1 diabetes development in non-obese diabetic mice. BMJ Open Diabetes Res. Care..

[78] Farkas H.S., Leschek E.W. (2025). Type 1 Diabetes Prevention: Screening Efforts and Prevention Studies in At-Risk Relatives and the General Population. Med. Res. Arch..

[79] Wentworth J.M., Oakey H., Craig M.E., Couper J.J., Cameron F.J., Davis E.A., Lafferty A.R., Harris M., Wheeler B.J., Jefferies C. (2022). Decreased occurrence of ketoacidosis and preservation of beta cell function in relatives screened and monitored for type 1 diabetes in Australia and New Zealand. Pediatr. Diabetes.

[80] Yu L., Herold K., Krause-Steinrauf H., McGee P.L., Bundy B., Pugliese A., Krischer J., Eisenbarth G.S. (2011). Type 1 Diabetes TrialNet Anti-CD20 Study Group. Rituximab selectively suppresses specific islet antibodies. Diabetes.

[81] Pescovitz M.D., Greenbaum C.J., Krause-Steinrauf H., Becker D.J., Gitelman S.E., Goland R., Gottlieb P.A., Marks J.B., McGee P.F., Moran A.M. (2009). Type 1 Diabetes TrialNet Anti-CD20 Study Group. Rituximab, B-lymphocyte depletion, and preservation of beta-cell function. N. Engl. J. Med..

[82] Urrutia I., Martinez R., Calvo B., Marcelo I., Saso-Jimenez L., Martinez de Lapiscina I., Bilbao J.R., Castano L., Rica I. (2024). Collaborative Working Group. Risk for progression to type 1 diabetes in first-degree relatives under 50 years of age. Front. Endocrinol..

[83] Besser R.E.J., Bell K.J., Couper J.J., Ziegler A.G., Wherrett D.K., Knip M., Speake C., Casteels K., Driscoll K.A., Jacobsen L. (2022). ISPAD Clinical Practice Consensus Guidelines 2022: Stages of type 1 diabetes in children and adolescents. Pediatr. Diabetes.

[84] Mahon J.L., Sosenko J.M., Rafkin-Mervis L., Krause-Steinrauf H., Lachin J.M., Thompson C., Bingley P.J., Bonifacio E., Palmer J.P., Eisenbarth G.S. (2009). TrialNet Natural History Committee; Type 1 Diabetes TrialNet Study Group. The TrialNet Natural History Study of the Development of Type 1 Diabetes: Objectives, design, and initial results. Pediatr. Diabetes.

[85] Krischer J.P., Schatz D.A., Bundy B., Skyler J.S., Greenbaum C.J., Writing Committee for the Type 1 Diabetes TrialNet Oral Insulin Study Group (2017). Effect of Oral Insulin on Prevention of Diabetes in Relatives of Patients with Type 1 Diabetes: A Randomized Clinical Trial. JAMA.

[86] (2002). Diabetes Prevention Trial--Type 1 Diabetes Study Group. Effects of insulin in relatives of patients with type 1 diabetes mellitus. N. Engl. J. Med..

[87] Frommer L., Kahaly G.J. (2020). Type 1 diabetes and associated autoimmune diseases. World J. Diabetes.

[88] Stahl M., Simmons K., Dong F., Felipe-Morales D., Liu E., Rewers M., ASK Study Group (2025). 2102-LB: The Autoimmunity Screening for Kids (ASK) Study Experience—Islet Autoimmunity Screening for Celiac Disease. Diabetes.

[89] Steck A.K., Dong F., Geno Rasmussen C., Bautista K., Sepulveda F., Baxter J., Yu L., Frohnert B.I., Rewers M.J. (2022). ASK Study Group CGM Metrics Predict Imminent Progression to Type 1 Diabetes: Autoimmunity Screening for Kids (ASK) Study. Diabetes Care.

[90] Calderone M., Aramnejad S., Giliberto E., Bombaci B., La Rocca M., Torre A., Lombardo F., Salzano G., Passanisi S. (2026). Early Detection of Pediatric Type 1 Diabetes: The Expanding Role of Screening. Children.

[91] Hattersley A.T., Patel K.A. (2017). Precision diabetes: Learning from monogenic diabetes. Diabetologia.

[92] Harris A.G., Letourneau L.R., Greeley S.A.W. (2018). Monogenic diabetes: The impact of making the right diagnosis. Curr. Opin. Pediatr..

[93] Quon J.C., Kaneta K., Fotiadis N., Menteer J., Lestz R.M., Weisert M., Baxter-Lowe L.A. (2023). HLA diversity in ethnic populations can affect detection of donor-specific antibodies by single antigen beads. Front. Immunol..

[94] Musoma S.N., Omar A., Mutai B.C., Laigong P. (2020). Outcomes of Children and Adolescents Admitted with Diabetic Ketoacidosis at Kenyatta National Hospital (KNH), Kenya. J. Diabetes Res..

[95] Murunga A.N., Owira P.M.O. (2013). Diabetic ketoacidosis: An overlooked child killer in sub-Saharan Africa?. Trop. Med. Int. Health.

[96] Vinay E.S., Laxmi Narsimha Rao B., Saf N., Gautam D. (2026). C-peptide in Precision Diabetes Care and Beyond: A Comprehensive Review. Clin. Med. Insights Endocrinol. Diabetes.

[97] Jones A.G., Hattersley A.T. (2013). The clinical utility of C-peptide measurement in the care of patients with diabetes. Diabet. Med..

[98] Wong T.W.C., Wong M.Y.S., But W.M.B. (2021). Features of partial remission in children with type 1 diabetes using the insulin dose-adjusted A1c definition and risk factors associated with nonremission. Ann. Pediatr. Endocrinol. Metab..

[99] Foteinopoulou E., Clarke C.A.L., Pattenden R.J., Ritchie S.A., McMurray E.M., Reynolds R.M., Arunagirinathan G., Gibb F.W., McKnight J.A., Strachan M.W.J. (2020). Impact of routine clinic measurement of serum C-peptide in people with a clinician-diagnosis of type 1 diabetes. Diabet. Med..

[100] Eason R.J., Thomas N.J., Hill A.V., Knight B.A., Carr A., Hattersley A.T., McDonald T.J., Shields B.M., Jones A.G., for the StartRight Study Group (2022). Routine islet autoantibody testing in clinically diagnosed adult-onset type 1 diabetes can help identify misclassification and the possibility of successful insulin cessation. Diabetes Care.

[101] Thomas N.J., Walkey H.C., Kaur A., Misra S., Oliver N.S., Colclough K., Weedon M.N., Johnston D.G., Hattersley A.T., Patel K.A. (2023). The relationship between islet autoantibody status and the genetic risk of type 1 diabetes in adult-onset type 1 diabetes. Diabetologia.

[102] Munoz C., Floreen A., Garey C., Karlya T., Jelley D., Alonso G.T., McAuliffe-Fogarty A. (2019). Misdiagnosis and diabetic ketoacidosis at diagnosis of type 1 diabetes: Patient and caregiver perspectives. Clin. Diabetes.

[103] Thomas N.J., Lynam A.L., Hill A.V., Weedon M.N., Shields B.M., Oram R.A., McDonald T.J., Hattersley A.T., Jones A.G. (2019). Type 1 diabetes defined by severe insulin deficiency occurs after 30 years of age and is commonly treated as type 2 diabetes. Diabetologia.

[104] Thakkar S., Chopra A., Nagendra L., Kalra S., Bhattacharya S. (2023). Teplizumab in Type 1 Diabetes Mellitus: An Updated Review. touchREV. Endocrinol..

[105] Saleem M.R., Khan M.T. (2025). Teplizumab: A promising intervention for delaying type 1 diabetes progression. Front. Endocrinol..

[106] Herold K.C., Bundy B.N., Long S.A., Bluestone J.A., DiMeglio L.A., Dufort M., Gitelman S.E., Gottlieb P.A., Krischer J.P., Linsley P.S. (2019). An anti-CD3 antibody, teplizumab, in relatives at risk for type 1 diabetes. N. Engl. J. Med..

[107] Herold K.C., Gitelman S.E., Gottlieb P.A., Knecht L.A., Raymond R., Ramos E.L. (2023). Teplizumab: A disease-modifying therapy for type 1 diabetes that preserves β-cell function. Diabetes Care.

[108] Galderisi A., Sims E.K., Evans-Molina C., Petrelli A., Cuthbertson D., Nathan B.M., Ismail H.M., Herold K.C., Moran A. (2025). Trajectory of beta cell function and insulin clearance in stage 2 type 1 diabetes: Natural history and response to teplizumab. Diabetologia.

[109] Lin C., Hu S., Cai X., Lv F., Yang W., Liu G., Yang X., Ji L. (2024). The opportunities and challenges of the disease-modifying immunotherapy for type 1 diabetes: A systematic review and meta-analysis. Pharmacol. Res..

[110] Hagopian W., Ferry R.J., Sherry N., Carlin D., Bonvini E., Johnson S., Stein K.E., Koenig S., Daifotis A.G., Herold K.C. (2013). Teplizumab preserves C-peptide in recent-onset type 1 diabetes: Two-year results from the randomized, placebo-controlled protégé trial. Diabetes.

[111] Ehlers M.R. (2016). Strategies for clinical trials in type 1 diabetes. J. Autoimmun..

[112] Pescovitz M.D., Greenbaum C.J., Bundy B., Becker D.J., Gitelman S.E., Goland R., Gottlieb P.A., Marks J.B., Moran A., Raskin P. (2014). Type 1 Diabetes TrialNet Anti-CD20 Study Group. B-lymphocyte depletion with rituximab and β-cell function: Two-year results. Diabetes Care.

[113] Zieliński M., Żalińska M., Iwaszkiewicz-Grześ D., Gliwiński M., Hennig M., Jaźwińska-Curyłło A., Kamińska H., Sakowska J., Wołoszyn-Durkiewicz A., Owczuk R. (2022). Combined therapy with CD4+ CD25highCD127- T regulatory cells and anti-CD20 antibody in recent-onset type 1 diabetes is superior to monotherapy: Randomized phase I/II trial. Diabetes Obes. Metab..

[114] Quattrin T., Haller M.J., Steck A.K., Felner E.I., Li Y., Xia Y., Leu J.H., Zoka R., Hedrick J.A., Rigby M.R. (2020). T1GER Study Investigators. Golimumab and Beta-Cell Function in Youth with New-Onset Type 1 Diabetes. N. Engl. J. Med..

[115] Rigby M.R., Hayes B., Li Y., Vercruysse F., Hedrick J.A., Quattrin T. (2023). Two-Year Follow-up from the T1GER Study: Continued Off-Therapy Metabolic Improvements in Children and Young Adults with New-Onset T1D Treated with Golimumab and Characterization of Responders. Diabetes Care.

[116] Greenbaum C.J., Serti E., Lambert K., Weiner L.J., Kanaparthi S., Lord S., Gitelman S.E., Wilson D.M., Gaglia J.L., Griffin K.J. (2021). ITN058AI EXTEND Study Team. IL-6 receptor blockade does not slow β cell loss in new-onset type 1 diabetes. JCI Insight.

[117] Piemonti L., Keymeulen B., Gillard P., Linn T., Bosi E., Rose L., Pozzilli P., Giorgino F., Cossu E., Daffonchio L. (2022). Ladarixin, an inhibitor of the interleukin-8 receptors CXCR1 and CXCR2, in new-onset type 1 diabetes: A multicentre, randomized, double-blind, placebo-controlled trial. Diabetes Obes. Metab..

[118] Kosmeri C., Klapas A., Evripidou N., Kantza E., Serbis A., Siomou E., Ladomenou F. (2025). Rotavirus Vaccination Protects Against Diabetes Mellitus Type 1 in Children in Developed Countries: A Systematic Review and Meta-Analysis. Vaccines.

[119] Rogers M.A., Basu T., Kim C. (2019). Lower incidence rate of type 1 diabetes after receipt of the rotavirus vaccine in the United States, 2001–2017. Sci. Rep..

[120] Doupis J., Kolokathis K., Markopoulou E., Efthymiou V., Festas G., Papandreopoulou V., Kallinikou C., Antikidou D., Gemistou G., Angelopoulos T. (2021). The role of pediatric BCG vaccine in type 1 diabetes onset. Diabetes Ther..

[121] Hyöty H., Kääriäinen S., Laiho J.E., Comer G.M., Tian W., Härkönen T., Lehtonen J.P., Oikarinen S., Puustinen L., Snyder M. (2024). Safety, tolerability and immunogenicity of PRV-101, a multivalent vaccine targeting coxsackie B viruses (CVBs) associated with type 1 diabetes: A double-blind randomised placebo-controlled Phase I trial. Diabetologia.

[122] Gupta S., Sharma T.K., Kaushik G.G., Shekhawat V.P.S. (2011). Vitamin E supplementation may ameliorate oxidative stress in type 1 diabetes mellitus patients. Clin. Lab..

[123] Asbaghi O., Nazarian B., Yousefi M., Anjom-Shoae J., Rasekhi H., Sadeghi O. (2023). Effect of vitamin E intake on glycemic control and insulin resistance in diabetic patients: An updated systematic review and meta-analysis of randomized controlled trials. Nutr. J..

[124] Ríos-Ríos W.J., Sosa-Luis S.A., Torres-Aguilar H. (2021). Current advances in using tolerogenic dendritic cells as a therapeutic alternative in the treatment of type 1 diabetes. World J. Diabetes.

[125] Jacobsen L.M., Schatz D.A. (2021). Insulin immunotherapy for pretype 1 diabetes. Curr. Opin. Endocrinol. Diabetes Obes..

[126] Nabi-Afjadi M., Ostadhadi S., Liaghat M., Pasupulla A.P., Masoumi S., Aziziyan F., Zalpoor H., Abkhooie L., Tarhriz V. (2024). Revolutionizing type 1 diabetes management: Exploring oral insulin and adjunctive treatments. Biomed. Pharmacother..

[127] Smith E.L., Peakman M. (2018). Peptide Immunotherapy for Type 1 Diabetes-Clinical Advances. Front. Immunol..

[128] Kumar D., Tanwar R., Gupta V. (2025). First-ever stem cell therapy restores insulin independence in type 1 diabetes: A medical milestone. World J. Stem Cells.

[129] Medenica S., Abazovic D., Vukovic J., Vojinovic T., Tomovic F., Defeudis G., Mazzilli R., Kovacevic Z., Djurdjic D., Prelevic V. (2026). Regenerative medicine approaches for treating diabetes: Current advances and future directions. J. Diabetes Complicat..

[130] Hogrebe N.J., Ishahak M., Millman J.R. (2023). Developments in stem cell-derived islet replacement therapy for treating type 1 diabetes. Cell Stem Cell.

[131] Han J., Lim D., Yang K. (2025). Gene editing strategies to address current challenges in stem cell-derived β cell therapy for type 1 Diabetes. J. Tissue Eng..

